# Uric Acid-to-HDL Cholesterol Ratio as an Independent Predictor of In-Hospital New-Onset Atrial Fibrillation in Non-ST-Elevation Myocardial Infarction

**DOI:** 10.3390/jcm15082977

**Published:** 2026-04-14

**Authors:** Mehmet Nail Bilen, Ömer Genç, Gazi Çapar, Muhammed Mert Göksu, Hüseyin Akgün, Gamze Acar, Göksenin Cansu Özdoğan, Günseli Üredi, Furkan Şen, Ufuk Sali Halil, Yusuf İnci, Deniz Dilan Naki Tekin, Fahri Er, Ersin İbişoğlu

**Affiliations:** İstanbul Başakşehir Çam ve Sakura Şehir Hastanesi, 34480 Istanbul, Turkey; dr.genc@hotmail.com (Ö.G.); gazicapar1@hotmail.com (G.Ç.); dr.mmgoksu@gmail.com (M.M.G.); husseyinakgun@gmail.com (H.A.); gamzeacar4747@gmail.com (G.A.); cansuozdogan03@gmail.com (G.C.Ö.); uredig@gmail.com (G.Ü.); fsfurkansen57@gmail.com (F.Ş.); oufoukchalil@gmail.com (U.S.H.); dryusufinci@gmail.com (Y.İ.); dilan_naki@hotmail.com (D.D.N.T.); bandirmaonyedieylul@hs01.kep.tr (F.E.); e_ibisoglu@hotmail.com (E.İ.)

**Keywords:** non-ST-elevation myocardial infarction, new-onset atrial fibrillation, uric acid-to-HDL cholesterol ratio, biomarker, inflammation

## Abstract

**Background:** To investigate the association between the uric acid-to-high-density lipoprotein cholesterol ratio (UAHDLr) and the risk of new-onset atrial fibrillation (NOAF) during hospitalization in patients with non-ST-elevation myocardial infarction (NSTEMI). **Methods:** This retrospective cohort study included NSTEMI patients without prior atrial fibrillation. UAHDLr was log2-transformed to evaluate the effect of doubling. Feature selection was performed using least absolute shrinkage and selection operator regression. Cox proportional hazards models with sequential adjustments were applied. Nonlinear associations were assessed using restricted cubic spline analysis, and discrimination was evaluated with time-dependent receiver operating characteristic analysis. **Results:** UAHDLr was independently associated with in-hospital NOAF across all models. Doubling (1-unit increase) UAHDLr significantly increased NOAF risk in unadjusted (HR: 5.97, 95% CI: 3.71–9.61) and clinically adjusted models (HR: 5.98, 95% CI: 3.58–9.99). The association was stronger in the LASSO-adjusted (HR: 8.19, 95% CI: 4.13–16.24) and fully adjusted models (HR: 13.40, 95% CI: 5.90–30.44). Spline analysis showed a progressive increase in NOAF risk with higher UAHDLr values. Discrimination was stable, with an area under the curve of approximately 0.76. **Conclusions:** UAHDLr is a strong, easily accessible biomarker for predicting in-hospital NOAF in NSTEMI patients and may support early risk stratification.

## 1. Introduction

Non-ST-elevation myocardial infarction (NSTEMI) is a common manifestation of acute coronary syndromes (ACS) and is associated with substantial morbidity and mortality worldwide. One clinically significant complication in patients with NSTEMI is new-onset atrial fibrillation (NOAF), which occurs in 5–20% of patients during hospitalization. NOAF is associated with worse outcomes, including stroke, heart failure, prolonged hospital stay, and increased mortality [1].

Despite advances in the acute management of NSTEMI, early identification of patients at high risk for NOAF remains challenging. Traditional risk factors, such as advanced age, left atrial enlargement, and heart failure, have limited predictive value. Therefore, there is a growing interest in identifying novel biomarkers that are easily accessible and cost-effective and reflect the underlying pathophysiological mechanisms, such as inflammation, oxidative stress, and endothelial dysfunction.

Uric acid (UA), the final product of purine metabolism, is increasingly recognized as a marker of oxidative stress and an independent risk factor for cardiovascular disease (CVD). A large Swedish cohort study found that elevated serum uric acid levels were significantly associated with an increased incidence of NOAF [2]. Similarly, including uric acid in the CHA_2_DS_2_-VASc score significantly improved the prediction of NOAF in patients with acute myocardial infarction [3].

Conversely, high-density lipoprotein cholesterol (HDL-C) has well-established anti-inflammatory and antioxidant properties and is inversely associated with cardiovascular risk. Recently, the uric acid-to-HDL cholesterol ratio (UAHDLr) has emerged as a composite biomarker of both oxidative stress and reduced lipid-related protection. Elevated UAHDLr has been associated with adverse cardiometabolic outcomes, including coronary artery disease, metabolic syndrome, and increased mortality [4,5].

The prognostic value of UAHDLr has been investigated in various cardiovascular diseases. However, its role in determining NOAF in patients with NSTEMI has not been adequately explored. Identifying patients at risk early using a simple biochemical marker, such as UAHDLr, could improve risk stratification and guide closer monitoring or preventive interventions in clinical practice.

The aim of this study was to investigate the association between UAHDLr levels and the risk of NOAF in patients hospitalized with NSTEMI. Our hypothesis was that elevated UAHDLr levels at the time of admission would be independently associated with a higher risk of NOAF and could serve as a practical prognostic marker for this patient population.

## 2. Methods

### 2.1. Ethics

This retrospective study was started after receiving approval from the Non-Interventional Clinical Research Ethics Committee of Başakşehir Çam and Sakura Hospital (Date: 17 December 2025, Decision No: KAEK/17.12.2025.448). It was performed in line with the principles of the Declaration of Helsinki.

### 2.2. Study Design and Population

This retrospective observational study included 988 consecutive patients diagnosed with NSTEMI and treated at our institution’s cardiology clinic between January 2023 and December 2025. According to the 2023 European Society of Cardiology ESC guidelines for managing acute coronary syndromes, all patients underwent coronary angiography during their initial hospitalization. Percutaneous coronary intervention (PCI) was performed on patients with hemodynamically significant coronary artery stenosis at the discretion of the interventional cardiologist treating them [6]. The primary outcome was the occurrence of NOAF during hospitalization. Time-to-event was defined as the length of the hospital stay (in days) from admission until the onset of NOAF or discharge, whichever occurred first.

Because of the retrospective design of the study, a formal a priori sample size calculation was not performed. Instead, all consecutive patients meeting the eligibility criteria during the study period (January 2023–December 2025) were included in the analysis to minimize selection bias and maximize statistical power. AI-assisted language editing was used to improve grammar, clarity, and readability; all scientific content, analyses, and final wording were reviewed and approved by the authors.

### 2.3. Inclusion and Exclusion Criteria

#### 2.3.1. Inclusion Criteria

Patients were eligible for inclusion if they met the following criteria:

1. Age ≥18 years;
2. A confirmed diagnosis of NSTEMI based on clinical presentation, cardiac biomarkers, and electrocardiographic findings;
3. Availability of serum uric acid and HDL-C levels at admission;
4. Follow-up and treatment were provided at our hospital’s cardiology clinic;
5. Ability to monitor the development of NOAF during hospitalization.

#### 2.3.2. Exclusion Criteria

Patients were excluded if they had any of the following conditions:

1. A previously known diagnosis of chronic atrial fibrillation was made.
2. The patient was diagnosed with ST-elevation myocardial infarction.
3. Patients with severe comorbid conditions such as renal failure, hepatic failure, or active malignancy.
4. Missing serum uric acid or HDL-C data.
5. Use of any antiarrhythmic medication other than beta- blockers prior to hospital admission.
6. Patients with significant electrolyte disturbances (including abnormal serum potassium, sodium, calcium, or magnesium levels) at admission were excluded to minimize the risk of arrhythmia attributable to electrolyte imbalance.

### 2.4. Definition of New-Onset Atrial Fibrillation

NOAF was defined as the first documented episode of atrial fibrillation occurring during hospitalization in patients without a prior history of atrial fibrillation. An AF episode was defined as an irregularly irregular rhythm without discernible P waves lasting ≥30 s on electrocardiographic recording [7].

### 2.5. Detection and Monitoring of NOAF

All patients underwent continuous cardiac telemetry monitoring during hospitalization. In addition, standard 12-lead electrocardiography (ECG) was performed at admission and whenever patients developed symptoms suggestive of arrhythmia, such as palpitations or hemodynamic instability. Telemetry recordings and ECG tracings were reviewed by experienced cardiologists to confirm the diagnosis of atrial fibrillation. The occurrence and timing of NOAF were documented using electronic medical records and monitoring system reports.

### 2.6. Data Collection

Demographic and clinical characteristics, cardiovascular risk factors, vital signs, and comorbidities were recorded from electronic medical records. Laboratory tests, including uric acid, HDL-C, complete blood count, renal and liver function tests, and inflammatory markers, were performed on venous blood samples collected within the first 24 h of admission. UAHDLr was calculated by dividing UA (mg/dL) by HDL-C (mg/dL). All patients underwent continuous cardiac monitoring during hospitalization and standard echocardiography within 48 h of admission.

### 2.7. Transthoracic Echocardiography (TTE)

Transthoracic echocardiography was performed in the left lateral decubitus position using a 3.25 MHz transducer (Vivid 5, GE Healthcare, Chicago, IL, USA). M-mode and two-dimensional (2D) echocardiographic images were obtained according to the recommendations of the American Society of Echocardiography [8]. Standard parasternal long- and short-axis views, as well as apical four- and two-chamber views, were used for cardiac measurements. The left ventricular ejection fraction was calculated using the modified Simpson’s biplane method [9].

### 2.8. Exposure Variable

The primary exposure of interest was the UAHDLr. Owing to its skewed distribution and extreme values, UAHDLr was log2-transformed [UAHDLr (log2)] before analysis. This transformation enabled the hazard ratios to be interpreted as the hazard ratio per doubling of the UAHDLr, thereby improving the interpretability and numerical stability of the models. Specifically, a 1-unit increase in UAHDLr (log2) corresponds to a doubling of the original UAHDLr value.

### 2.9. Feature Selection

**Figure 1:**
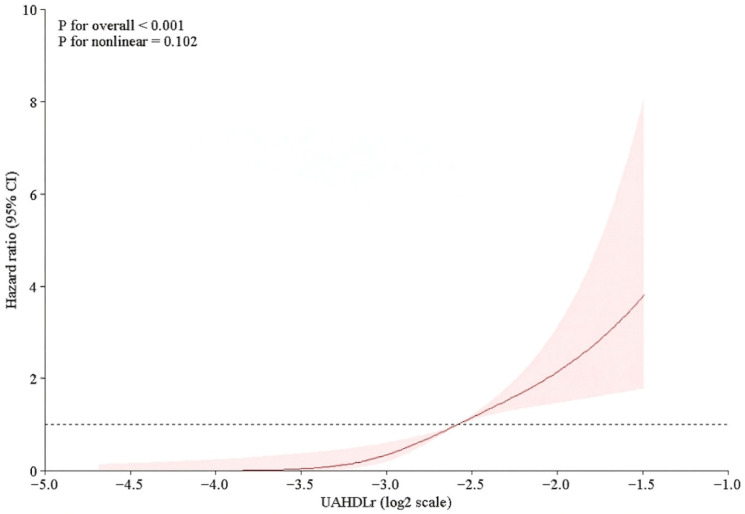
Restricted cubic spline illustrating the association between UAHDLr (log2) and risk of in-hospital NOAF.

To identify variables potentially associated with in-hospital NOAF while avoiding overfitting in a high-dimensional dataset, the Least Absolute Shrinkage and Selection Operator (LASSO) regression method was used for feature selection (Figure 1). LASSO was used in an exploratory manner to identify candidate predictors among demographic, clinical, laboratory, and procedural variables. Variables with non-zero coefficients were retained for subsequent modeling.

## 3. Statistical Analysis

Continuous variables were subjected to normality distribution analyses using an analytical method (Kolmogorov–Smirnov test) and visual methods (histograms and probability plots). Continuous variables are presented as mean ± standard deviation or median (interquartile range; IQR25-75), as appropriate. Categorical variables are presented as counts and percentages.

Continuous variables were compared using the independent Student’s *t*-test or Mann–Whitney U-test, while the chi-square test or Fisher’s exact test was used to compare categorical variables.

Time-to-event analyses were performed using Cox proportional hazards regression, with in-hospital NOAF as the outcome and hospital stay duration as the time scale. The association between UAHDLr (log2) and incident NOAF was evaluated using a sequential modeling strategy to assess the robustness of the findings across different adjustment models.

### Four Cox Models Were Constructed

Model 1 (unadjusted): Included UAHDLr (log2) only.

Model 2 (clinically adjusted): Adjusted for established clinical risk factors for atrial fibrillation, including age, body mass index, hypertension, diabetes mellitus, heart failure, chronic obstructive pulmonary disease, prior coronary artery bypass grafting, prior coronary angiography, prior coronary stenting, and left ventricular ejection fraction.

Model 3 (LASSO—selected model): Included UAHDLr (log2) and variables identified through LASSO feature selection.

Model 4 (fully adjusted model): Combination of clinically relevant covariates and LASSO-selected variables.

Hazard ratios (HRs) with 95% confidence intervals (CIs) were reported for the analyses. For clarity, only the HRs for UAHDLr (log2) are presented in the main table, while the full model coefficients are available in the Supplementary Materials (Supplementary Table S1). The proportional hazards assumption was evaluated using Schoenfeld residuals for the unadjusted and clinically adjusted models, with no significant violations observed (global test *p*-values > 0.05). For models with a large number of covariates, where Schoenfeld residual-based testing was not numerically stable, we assessed the proportional hazards assumption for the primary exposure [UAHDLr (log2) using time-interaction terms. These analyses did not demonstrate any significant time-dependent effects (*p* > 0.05), supporting the validity of the proportional hazards assumption.

To explore the potential non-linear associations between UAHDLr (log2) and the risk of in-hospital NOAF, restricted cubic spline (RCS) analysis was performed within the Cox regression framework. Three knots were placed at the predefined percentiles of the UAHDLr (log2) distribution. Hazard ratios were plotted relative to a clinically meaningful reference value to allow visualization of the dose–response relationship.

The optimal cutoff value of UAHDLr (log2) for identifying in-hospital NOAF was determined using the maximally selected rank statistics (Figure 1). Based on this cutoff, patients were categorized into low and high UAHDLr groups, respectively. Additionally, quantile-based analyses and ROC-derived performance metrics were evaluated to complement cutoff-based findings. Kaplan–Meier curves were generated to compare NOAF-free survival between the two groups, and the log-rank test was used to assess the differences. The numbers at risk are displayed beneath the survival curves.

We evaluated the discriminative performance of UAHDLr (log2) for determining in-hospital NOAF over time using time-dependent receiver operating characteristic (ROC) curve analysis. Time-dependent area under the curve (AUC) values were estimated and plotted to evaluate the predictive performance stability throughout hospitalization. All statistical analyses were performed using R software (version 4.3.2). A two-sided *p*-value of less than 0.05 was considered statistically significant.

## 4. Results

### 4.1. Baseline Characteristics According to In-Hospital Atrial Fibrillation

The baseline demographic, clinical, laboratory, and procedural characteristics of the 988 patients included in the study, stratified by the occurrence of NOAF, are presented in Table 1. Patients who developed NOAF (50 individuals-5.1%) during hospitalization were older and had a higher body mass index (BMI) than those who remained in sinus rhythm. In addition, several comorbidities, including heart failure, prior stroke, and chronic kidney failure, were more prevalent in patients who developed NOAF.

In terms of laboratory parameters, patients diagnosed with in-hospital NOAF demonstrated lower levels of hemoglobin, lymphocytes, low-density lipoprotein, and total cholesterol at the time of admission. Similarly, patients with in-hospital NOAF demonstrated a reduced left ventricular ejection fraction. Conversely, former NOAF patients exhibited an elevated UAHDLr (log2) in comparison to those who did not develop NOAF (−2.19 ± 0.47 vs. −2.98 ± 0.65, *p* < 0.001).

The length of hospital stay was similar between the groups [3 (2–7) days vs. 4 (2–9) days, *p* = 0.11]. The average time to atrial fibrillation onset among NOAF patients was 48 h.

In-hospital mortality rates were also comparable between patients with and without NOAF [2 (4.0%) vs. 28 (3.0%), *p* = 0.68].

### 4.2. Association Between UAHDLr (log2) and In-Hospital NOAF

#### 4.2.1. Cox Proportional Hazards Regression

The association between UAHDLr and in-hospital NOAF remained robust across all the Cox regression models (Table 2). In the unadjusted model, each doubling (a 1-unit increase) of UAHDLr was associated with a nearly six-fold increased risk of in-hospital NOAF (HR 5.97, 95% CI 3.71–9.61; *p* < 0.001). This association persisted after adjusting for established clinical NOAF risk factors (Model 2: HR 5.98, 95% CI 3.58–9.99; *p* < 0.001). Adjusting for the LASSO-selected variables further strengthened this association (Model 3: HR 8.19, 95% CI 4.13–16.24; *p* < 0.001). In the fully adjusted model combining clinical risk factors and LASSO-selected variables, UAHDLr remained a powerful independent determinant of NOAF, with a more than 13-fold increase in risk per doubling of UAHDLr (Model 4: HR 13.40, 95% CI 5.90–30.44; *p* < 0.001).

#### 4.2.2. Non-Linear Association Between UAHDLr (log2) and NOAF Risk

Restricted cubic spline analysis revealed a nonlinear dose–response relationship between UAHDLr (log2) and the risk of in-hospital NOAF. The risk of NOAF increased progressively with higher UAHDLr (log2) levels, and there was no clear evidence of a plateau effect. This pattern remained consistent after adjusting for the relevant covariates. The spline curve with 95% confidence intervals is presented in Figure 1.

#### 4.2.3. Optimal Cutoff Value and Risk Stratification

Using maximally selected rank statistics, an optimal cutoff value of −2.31 for UAHDLr (log2) was identified. Based on this cutoff, patients were categorized into low and high UAHDLr groups.

#### 4.2.4. Kaplan–Meier Analysis According to UAHDLr Groups

**Figure 2:**
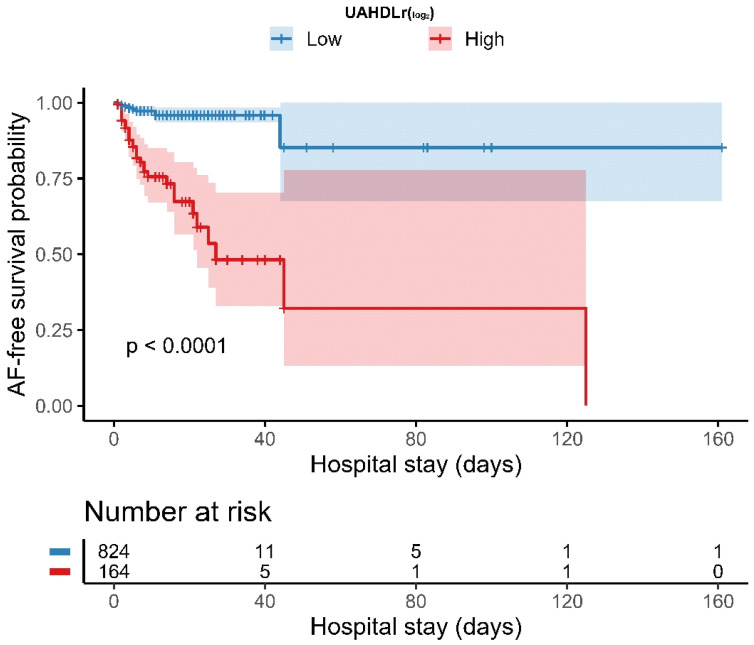
Kaplan–Meier curves for NOAF-free survival stratified by UAHDLr (log2) groups.

Kaplan–Meier analysis demonstrated a significantly lower NOAF-free survival probability in patients with a high UAHDLr (log2) than in those with a low UAHDLr (log2). The divergence between the survival curves occurred early during hospitalization and persisted throughout the follow-up period (log-rank *p* < 0.001). The NOAF-free survival curves along with the numbers at risk are presented in Figure 2.

#### 4.2.5. Time-Dependent Discriminative Performance of UAHDLr (log2)

**Figure 3:**
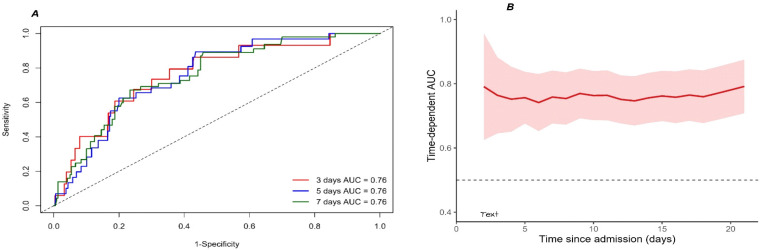
Time-dependent receiver operating characteristic (ROC) analysis of the uric acid-to-HDL cholesterol ratio (UAHDLr, log2-transformed) for the prediction of in-hospital NOAF. (**A**) ROC curves and corresponding area under the curve (AUC) values for UAHDLr at 3, 5, and 7 days after admission. (**B**) Time-dependent AUC demonstrating the discriminative performance of UAHDLr over the course of hospitalization, with the shaded area indicating the 95% confidence interval. The horizontal dashed line represents an AUC of 0.5.

Time-dependent ROC analysis demonstrated a stable and consistent discriminative performance of UAHDLr across clinically relevant time points (Figure 3A,B). The AUC values at 3, 5, and 7 days were all approximately 0.76, indicating good and sustained predictive ability during early hospitalization. The time-dependent AUC remained relatively stable over time, with no evidence of performance attenuation, supporting the robustness of UAHDLr as a time-sensitive prognostic marker.

In quartile-based analyses, a graded increase in NOAF risk was observed across increasing UAHDLr categories (Supplementary Table S2), with the highest quartile showing a markedly elevated risk compared to the lowest. ROC analysis identified an optimal UAHDLr (log2) threshold of −2.72, corresponding to a sensitivity of 0.92 and a specificity of 0.66.

## 5. Discussion

In this retrospective cohort study of patients hospitalized with NSTEMI, we demonstrated that UAHDLr is a strong independent determinant of NOAF during hospitalization. The association between UAHDLr and in-hospital NOAF remained consistent across multiple analytical strategies, including unadjusted, clinically adjusted, least absolute shrinkage and selection operator-selected, and fully adjusted Cox proportional hazards models. These findings were robust when the UAHDLr was analyzed as a continuous variable after log2 transformation, which allowed us to interpret the risk per doubling of the ratio. They were also robust when we explored potential nonlinear effects using restricted cubic spline analysis.

A key methodological strength of the present study is that it explicitly considered the duration of hospitalization (days) as the time scale in survival analyses. In patients with NSTEMI, NOAF most frequently occurs during the initial phase of hospitalization when the ischemic burden, sympathetic activation, systemic inflammation, and oxidative stress are at their highest. By modeling time-to-event as days from admission to NOAF onset or discharge, our analysis captured the clinically relevant and dynamic window during which arrhythmic risk was highest. The early separation of NOAF-free survival curves in the Kaplan–Meier analysis further supports the idea that UAHDLr identifies patients who are vulnerable to early in-hospital arrhythmogenesis rather than merely reflecting long-term susceptibility.

NOAF is a common complication of acute coronary syndromes, with reported in-hospital incidence rates ranging from 5% to 20%, especially among patients with NSTEMI [10,11]. NOAF occurrence has been consistently associated with adverse outcomes, including ischemic stroke, acute heart failure, prolonged length of stay, and increased short- and long-term mortality [12,13]. Traditional predictors, such as advanced age, left atrial enlargement, reduced left ventricular ejection fraction, and heart failure, are well established; however, their discriminative performance remains modest and insufficient to fully explain NOAF development in the acute ischemic setting [14,15]. This underscores the need for biomarkers that reflect the acute pathophysiological milieu of NSTEMI, rather than static baseline risk alone.

Uric acid, the final product of purine metabolism, has emerged as an active contributor to cardiovascular pathology rather than a passive metabolic byproduct. Elevated uric acid levels are closely linked to increased xanthine oxidase activity, oxidative stress, endothelial dysfunction, and systemic inflammation. Large population-based studies, including the Swedish AMORIS cohort, have demonstrated a significant association between elevated serum uric acid levels and NOAF onset [2,16,17]. Furthermore, experimental data suggest that uric acid can promote atrial electrical and structural remodeling by activating proinflammatory pathways, including the NLRP3 inflammasome. This process facilitates NOAF initiation and maintenance [18].

In the context of acute myocardial infarction, oxidative stress and inflammatory activation are markedly amplified, particularly during the initial days of hospitalization. Previous studies have shown that incorporating uric acid into established clinical risk scores, such as the CHA_2_DS_2_-VASc score, improves the prediction of NOAF after myocardial infarction [3]. Our findings build on this evidence by showing that when considered alongside HDL-C as a composite marker, the association between UA and early in-hospital NOAF becomes even stronger.

Conversely, HDL-C exerts multiple cardioprotective effects, including antioxidant, anti-inflammatory, antithrombotic, and endothelial stabilizing properties [19]. Low HDL-C levels are associated with a greater inflammatory burden and poorer outcomes in patients with coronary artery disease and acute coronary syndromes [20]. The UAHDLr combines these opposing biological processes, excess oxidative stress and diminished antioxidant defense, into one metric, providing a more complete picture of the proarrhythmic environment during acute ischemia.

In addition to the oxidative and inflammatory pathways, there is growing evidence that acute disturbances in glucose metabolism also play an important role in the pathophysiology and prognosis of NSTEMI. Recent studies have shown that indices of stress hyperglycemia reflecting acute metabolic dysregulation are associated with adverse peri-procedural outcomes. For instance, a recent study examining the impact of glucometabolic status on type 4a myocardial infarction revealed that an elevated stress hyperglycemia ratio was an independent predictor of an increased risk of periprocedural myocardial injury and microvascular complications in patients with NSTEMI undergoing PCI [21]. These findings support the concept that acute metabolic stress interacts with inflammatory and oxidative pathways to influence cardiovascular outcomes in the setting of acute coronary syndromes. In this broader context, UAHDLr may represent a complementary biomarker within a ‘glucometabolic–inflammatory’ risk-stratification framework. By integrating information related to oxidative burden (uric acid) and reduced anti-inflammatory/antioxidant capacity (low HDL-C), UAHDLr may capture a pathophysiological milieu similar to that reflected by stress hyperglycemia-based indices. This further supports its potential value in identifying high-risk NSTEMI patients during the vulnerable in-hospital period.

Several pathophysiological mechanisms may explain the strong association between elevated UAHDLr and NOAF. Oxidative stress can induce apoptosis in atrial myocytes, disrupt intracellular calcium handling, and promote conduction abnormalities. These effects facilitate the initiation and perpetuation of NOAF. Endothelial dysfunction, which is often seen in states of hyperuricemia and low HDL-C levels, leads to microvascular ischemia, impaired atrial perfusion, and local inflammatory activation. Inflammatory signaling pathways, particularly those involving the NLRP3 inflammasome and interleukin-6, are strongly associated with NOAF pathogenesis and may serve as a link between elevated UAHDLr and arrhythmic susceptibility [18,22].

Several studies have linked elevated UAHDLr levels to adverse cardiometabolic and cardiovascular outcomes, including metabolic syndrome, insulin resistance, coronary artery disease severity, and increased mortality [22,23,24,25]. However, data on its role in predicting NOAF during NSTEMI hospitalization are scarce. Our study addresses this gap by demonstrating a strong, dose-dependent association between UAHDLr and NOAF risk during hospitalization. Restricted cubic spline analysis revealed a progressive increase in NOAF risk with increasing UAHDLr levels. No evidence of a clear threshold or plateau was observed, suggesting a continuous biological gradient.

**Figure 4:**
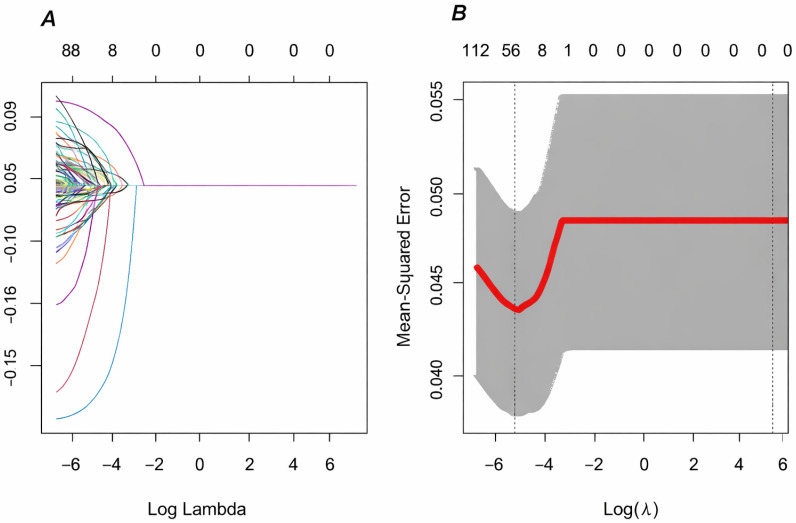
Least absolute shrinkage and selection operator (LASSO) regression analysis for variable selection. (**A**) Ten-fold cross-validation curve showing the optimal λ minimizing mean squared error. (**B**) LASSO coefficient profiles; non-zero coefficients at selected λ were retained for multivariable Cox models.

The robustness of this association was further supported by the LASSO-based feature selection process shown in Figure 4. LASSO regression identified the most informative predictors from a large set of clinical and laboratory variables while minimizing overfitting. Notably, UAHDLr retained a non-zero coefficient at the optimal penalty parameter and remained strongly associated with NOAF risk in subsequent Cox models. This indicates that UAHDLr provides prognostic information beyond that captured by traditional risk factors. From a temporal perspective, the stable time-dependent AUC values observed across clinically relevant in-hospital days (approximately 0.76 on days 3, 5, and 7) suggest that UAHDLr maintains its discriminative ability throughout the early and intermediate phases of hospitalization. This finding is particularly relevant given the dynamic evolution of arrhythmic risk in patients with NSTEMI, supporting the use of UAHDLr for time-to-event-based risk assessment rather than single-time-point prediction.

NOAF in patients with NSTEMI predominantly occurs during the early in-hospital period when ischemia-related inflammation and oxidative stress are most intense. In this context, our findings reveal that UAHDLr is a powerful and easily accessible biomarker that may identify the development of NOAF during hospital stay. Therefore, UAHDLr measurement at admission may enable the early identification of patients at the highest arrhythmic risk during the most vulnerable phase of hospitalization. Because UAHDLr reflects both heightened oxidative burden and reduced anti-inflammatory protection, it provides mechanistic insight in addition to prognostic value. Patients with elevated UAHDLr levels may benefit from intensified rhythm monitoring and early preventive strategies. The strong, stable, and time-dependent performance of this marker emphasizes its relevance in dynamic time-to-event-based risk assessment. Future prospective studies should determine whether modulating oxidative stress can reduce in-hospital NOAF and improve clinical outcomes.

### Study Limitations

First, the retrospective observational design precludes establishing a causal relationship between UAHDLr and in-hospital NOAF. Second, UAHDLr was measured only at admission; therefore, dynamic changes in uric acid and HDL-C levels during hospitalization and the potential prognostic value of serial measurements could not be evaluated. Third, although multivariable adjustment and LASSO-based feature selection were applied to reduce confounding and overfitting, residual confounding cannot be completely excluded. Renal function and in-hospital pharmacological management may have influenced both UAHDLr levels and the risk of NOAF. Although renal function was adjusted for using estimated glomerular filtration rate and baseline creatinine, unmeasured aspects of renal impairment and variations in medication use (e.g., diuretics, urate-lowering agents, statins, or anti-inflammatory drugs) may still have affected UAHDLr values. Furthermore, because UAHDLr was measured only at admission, reverse causality related to acute metabolic changes cannot be entirely ruled out. Fourth, this was a single-center study, which may limit the generalizability of the findings. Fifth, although continuous cardiac monitoring was performed during hospitalization, asymptomatic or very brief NOAF episodes may have been missed, potentially leading to an underestimation of the true incidence. Sixth, although left atrial size and mitral regurgitation are recognized as important echocardiographic parameters, they could not be included in the main analyses due to the retrospective design of the study and the high proportion of missing data (>50%). The inability to incorporate these variables may limit a more comprehensive assessment of structural cardiac factors associated with NOAF risk. Therefore, the results should be interpreted with caution in terms of potential residual bias. Future prospective studies with more complete echocardiographic data may provide more robust insights into these associations.

Finally, although the proportional hazards assumption was assessed and no significant violations were detected, the risk of NOAF during hospitalization may vary over time in the acute clinical setting. Therefore, potential time-varying effects cannot be completely excluded and should be further investigated in prospective studies with repeated temporal assessments.

## 6. Conclusions

This study found that UAHDLr is independently associated with the development of in-hospital NOAF in patients with NSTEMI. Higher UAHDLr levels at admission were associated with an increased risk of NOAF. This suggests a potential link between oxidative stress and atrial arrhythmogenesis in this population.

However, due to the retrospective design of the study and its inherent limitations, including the absence of detailed angiographic characteristics (e.g., lesion complexity and time to PCI), these findings should be interpreted with caution. Therefore, rather than establishing causality, our results should be considered hypothesis-generating.

From a clinical perspective, UAHDLr may represent an easy-to-measure biomarker that could aid in early risk stratification. Nevertheless, its incremental value over established clinical and echocardiographic predictors requires further investigation.

Future prospective, multicenter studies with comprehensive clinical and procedural data are needed to validate these findings, better define UAHDLr’s role in predicting NOAF, and explore whether reducing oxidative stress could lower NOAF incidence.

## Figures and Tables

**Table 1:** Baseline characteristics of the study population according to the occurrence of NOAF.

Variable	AF Absent (*n* = 938)	AF Present (*n* = 50)	*p*-Value	SMD
Clinical characteristics				
Age, years	60.86 ± 12.28	68.46 ± 13.03	<0.001	0.60
Male sex, *n* (%)	676 (72.1)	27 (54.0)	0.01	0.38
BMI, kg/m^2^	28.16 ± 4.89	30.05 ± 6.30	0.09	0.34
Heart rate, bpm	76.43 ± 13.35	76.48 ± 22.16	0.98	0.03
Systolic blood pressure, mmHg	124.71 ± 19.57	125.40 ± 17.78	0.81	0.04
Diastolic blood pressure, mmHg	72.85 ± 11.66	70.60 ± 15.45	0.19	0.16
Length of stay, days	3 (2–7)	4 (2–9)	0.11	0.22
Chest pain characteristics			0.04	0.40
None, *n* (%)	27 (2.9)	2 (4.0)		
Typical, *n* (%)	663 (70.7)	26 (52.0)		
Atypical, *n* (%)	173 (18.4)	14 (28.0)		
Uncertain, *n* (%)	75 (8.0)	8 (16.0)		
Dyspnea, *n* (%)	164 (17.5)	18 (36.0)	0.02	0.43
Palpitations, *n* (%)	43 (4.6)	12 (24.0)	<0.001	0.58
CAG history, *n* (%)			0.17	0.05
Normal	95 (9.6)	4 (8.0)		
Non-obstructive	311 (31.4)	10 (20.0)		
Obstructive	582 (59.0)	36 (72.0)		
Hypertension, *n* (%)	622 (66.3)	37 (74.0)	0.33	0.17
Diabetes mellitus, *n* (%)	421 (44.9)	25 (50.0)	0.57	0.10
Prior stroke, *n* (%)	56 (6.0)	7 (14.0)	0.049	0.27
Heart failure, *n* (%)	177 (18.9)	19 (38.0)	0.02	0.43
COPD, *n* (%)	72 (7.7)	7 (14.0)	0.18	0.20
Chronic kidney disease, *n* (%)	116 (12.4)	12 (24.0)	0.03	0.31
In-hospital mortality, *n* (%)	28 (3.0)	2 (4.0)	0.68	0.06
Laboratory findings				
Hemoglobin, g/dL	13.25 ± 2.19	12.00 ± 2.41	<0.001	0.54
White blood cells, ×10^9^/L	9.06 [7.29–11.35]	8.84 [6.84–11.14]	0.47	0.17
Lymphocytes, ×10^9^/L	2.07 [1.47–2.78]	1.79 [1.21–2.41]	0.03	0.20
Neutrophils, ×10^9^/L	6.50 ± 3.07	6.39 ± 2.78	0.79	0.04
Platelets, ×10^9^/L	248 [206–295]	239 [220–309]	0.72	0.18
Creatinine, mg/dL	1.11 ± 0.84	1.28 ± 0.93	0.16	0.20
eGFR, mL/min/1.73 m^2^	78.11 ± 26.82	64.48 ± 28.32	0.01	0.49
Uric acid, mg/dL	5.19 ± 1.60	8.04 ± 1.31	<0.001	1.95
Uric acid-to-HDL ratio (original)	0.14 ± 0.06	0.23 ± 0.08	<0.001	1.30
Uric acid-to-HDL ratio (log2)	−2.98 ± 0.65	−2.19 ± 0.47	<0.001	1.40
C-reactive protein, mg/L	5.75 [2.20–17.00]	11.00 [3.22–30.00]	0.02	0.23
Total cholesterol, mg/dL	182.27 ± 47.78	155.14 ± 43.04	<0.001	0.60
LDL cholesterol, mg/dL	111.34 ± 41.01	91.96 ± 34.65	0.01	0.51
HDL cholesterol, mg/dL	40.73 ± 13.32	37.54 ± 10.58	0.10	0.27
Triglycerides, mg/dL	132 [91–194]	116 [91–165]	0.23	0.16
Troponin and echocardiography				
Troponin at admission, ng/L	58.00 [23.10–189.75]	74.40 [25.93–327.75]	0.16	0.20
Peak troponin, ng/L	222.50 [55.00–771.75]	302.00 [80.25–1070.25]	0.34	0.15
Left ventricular ejection fraction, %	51.31 ± 11.06	46.58 ± 12.85	0.004	0.40

Abbreviations: Values are presented as mean ± standard deviation, median [interquartile range], or number (%), as appropriate. *p* values were calculated using Student’s *t*-test, Mann–Whitney *U* test, or χ^2^ test, as appropriate. SMD indicates standardized mean difference; values > 0.30 suggest clinically relevant imbalance. AF, atrial fibrillation; BMI, body mass index; BP, blood pressure; CAD, coronary artery disease; CAG, coronary angiography; CKD, chronic kidney disease; COPD, chronic obstructive pulmonary disease; CRP, C-reactive protein; eGFR, estimated glomerular filtration rate; HDL, high-density lipoprotein; LDL, low-density lipoprotein; PCI, percutaneous coronary intervention.

**Table 2:** Association between UAHDLr (log2) and in-hospital NOAF across sequential Cox proportional hazards models.

Model	HR	95% CI	*p*-Value
Model 1: Unadjusted	5.97	3.71–9.61	<0.001
Model 2: Clinical risk factors	5.98	3.58–9.99	<0.001
Model 3: LASSO-selected variables	8.19	4.13–16.24	<0.001
Model 4: Clinical + LASSO	13.40	5.90–30.44	<0.001

Abbreviations: Model 1 included UAHDLr (log2) only; Model 2 included UAHDLr (log2), age, body mass index, hypertension, diabetes mellitus, heart failure, chronic obstructive pulmonary disease, prior coronary artery bypass grafting, prior stent implantation, history of coronary angiography, and left ventricular ejection fraction; Model 3 included variables selected by least absolute shrinkage and selection operator (LASSO) regression. Model 4 combined variables from Models 2 and 3; UAHDLr indicates uric acid to high-density lipoprotein cholesterol ratio; HR, hazard ratio; CI, confidence interval.

## Data Availability

The data supporting this study are available from the corresponding author upon reasonable request.

## Supplementary Materials

The following supporting information can be downloaded at: https://www.mdpi.com/article/10.3390/jcm15082977/s1, Table S1: Association Between UAHDLr (log2) and In-Hospital Atrial Fibrillation Across Sequential Cox Proportional Hazards Models. Abbreviations: UAHDLr, uric acid–to–HDL cholesterol ratio; BMI, body mass index; LVEF, left ventricular ejection fraction; CABG, coronary artery bypass grafting; CAG, coronary angiography; ECG, electrocardiography; OM, obtuse marginal coronary artery; COPD, chronic obstructive pulmonary disease; Table S2: Association between UAHDLr (log2) quartiles and in-hospital new-onset atrial fibrillation (NOAF) in patients with NSTEMI, along with ROC-derived performance metrics. Legend: ROC Analysis Optimal threshold (log2 UAHDLr): −2.72; Sensitivity: 0.92; Specificity: 0.66.
