# Supplementary material for: Uric Acid-to-HDL Cholesterol Ratio as an Independent Predictor of In-Hospital New-Onset Atrial Fibrillation in Non-ST-Elevation Myocardial Infarction
Source: J Clin Med. 2026 Apr 14;15(8):2977. doi: 10.3390/jcm15082977 (PMC13117371; doi:10.3390/jcm15082977)
Supplement: Supplementary file 1 [file jcm-15-02977-s001.zip › Supplementary Table S1.pdf]

Supplementary Table S1. Association Between UAHDLr (log2) and In-Hospital Atrial Fibrillation Across Sequential Cox Proportional Hazards Models

Model	Variable	HR	95% CI (Lower)	95% CI (Upper)	p value
Model 1: Unadjusted	UAHDLr (log2)	5.97	3.71	9.61	<0.001
Model 2: Clinical risk factors	UAHDLr (log2)	5.98	3.58	9.99	<0.001
Model 2: Clinical risk factors	Age	1.04	1.01	1.06	0.006
Model 2: Clinical risk factors	BMI	1.06	1.01	1.12	0.019
Model 2: Clinical risk factors	Hypertension	0.76	0.38	1.52	0.434
Model 2: Clinical risk factors	Diabetes mellitus	0.62	0.34	1.13	0.119
Model 2: Clinical risk factors	Heart failure	1.46	0.65	3.28	0.359
Model 2: Clinical risk factors	COPD	1.64	0.70	3.86	0.258
Model 2: Clinical risk factors	CABG history	0.81	0.33	1.99	0.649
Model 2: Clinical risk factors	Prior CAG	1.06	0.73	1.53	0.758
Model 2: Clinical risk factors	Prior stent	1.53	0.84	2.80	0.167
Model 2: Clinical risk factors	LVEF	1.03	1.00	1.06	0.090
Model 3: LASSO-selected	UAHDLr (log2)	8.19	4.13	16.24	<0.001
Model 3: LASSO-selected	Age	1.01	0.98	1.04	0.668
Model 3: LASSO-selected	Sex	1.95	0.73	5.16	0.180
Model 3: LASSO-selected	BMI	1.08	1.00	1.16	0.041
Model 3: LASSO-selected	Diastolic BP	0.99	0.97	1.02	0.526
Model 3: LASSO-selected	Symptoms	1.38	0.85	2.23	0.189
Model 3: LASSO-selected	Dyspnea	0.82	0.37	1.80	0.615
Model 3: LASSO-selected	Palpitations	14.59	5.20	40.99	<0.001
Model 3: LASSO-selected	ECG abnormality	1.75	1.11	2.76	0.016
Model 3: LASSO-selected	Cerebrovascular disease	0.98	0.32	2.98	0.966
Model 3: LASSO-selected	Hyperlipidemia	0.37	0.18	0.76	0.007
Model 3: LASSO-selected	COVID-19 vaccination	0.15	0.05	0.43	<0.001
Model 3: LASSO-selected	Intubation requirement	0.10	0.01	0.70	0.020
Model 3: LASSO-selected	Diagonal lesion	0.27	0.08	0.85	0.026
Model 4: Clinical + LASSO	UAHDLr (log2)	13.40	5.90	30.44	<0.001
Model 4: Clinical + LASSO	Palpitations	13.23	4.68	37.45	<0.001
Model 4: Clinical + LASSO	ECG abnormality	1.88	1.15	3.07	0.012
Model 4: Clinical + LASSO	Hyperlipidemia	0.36	0.16	0.81	0.014
Model 4: Clinical + LASSO	COVID-19 vaccination	0.17	0.06	0.51	0.001
Model 4: Clinical + LASSO	Intubation requirement	0.11	0.01	0.81	0.030
Model 4: Clinical + LASSO	Diagonal lesion	0.23	0.06	0.86	0.029
Model 4: Clinical + LASSO	OM lesion	2.79	1.09	7.17	0.033
Model 4: Clinical + LASSO	Hypertension	0.27	0.09	0.79	0.016
Model 4: Clinical + LASSO	COPD	4.69	1.55	14.15	0.006
Model 4: Clinical + LASSO	CABG history	8.05	1.57	41.35	0.013
Model 4: Clinical + LASSO	LVEF	1.04	1.00	1.09	0.076

Abbreviations: UAHDLr, uric acid–to–HDL cholesterol ratio; BMI, body mass index; LVEF, left ventricular ejection fraction; CABG, coronary artery bypass grafting; CAG, coronary angiography; ECG, electrocardiography; OM, obtuse marginal coronary artery**;** COPD, chronic obstructive pulmonary disease.
