# Supplementary material for: Uric Acid-to-HDL Cholesterol Ratio as an Independent Predictor of In-Hospital New-Onset Atrial Fibrillation in Non-ST-Elevation Myocardial Infarction
Source: J Clin Med. 2026 Apr 14;15(8):2977. doi: 10.3390/jcm15082977 (PMC13117371; doi:10.3390/jcm15082977)
Supplement: Supplementary file 1 [file jcm-15-02977-s001.zip › Supplementary Table S2.pdf]

Supplementary Table S2

Association between UAHDLr (log2) quartiles and in-hospital new-onset atrial fibrillation (NOAF) in patients with NSTEMI, along with ROC-derived performance metrics.

UAHDLr Quartile	Hazard Ratio (HR)	95% CI (approx)	p-value
Q1 (Reference)	1.00	-	-
Q2	2.63	Wide CI	0.402
Q3	6.49	Wide CI	0.078
Q4	26.69	Wide CI	0.001

ROC Analysis

Optimal threshold (log_2 UAHDLr): -2.72

Sensitivity: 0.92

Specificity: 0.66
